# Possible activation of NRF2 by Vitamin E/Curcumin against altered thyroid hormone induced oxidative stress via NFĸB/AKT/mTOR/KEAP1 signalling in rat heart

**DOI:** 10.1038/s41598-019-43320-5

**Published:** 2019-05-15

**Authors:** Pallavi Mishra, Biswaranjan Paital, Srikanta Jena, Shasank S. Swain, Sunil Kumar, Manoj K. Yadav, Gagan B. N. Chainy, Luna Samanta

**Affiliations:** 1 Department of Zoology, Utkal University, Bhubaneswar, 751004 India; 2 Department of Zoology, Government Autonomous College, Phulbani, Kandhamal, Odisha India; 3 Redox Regulation Laboratory, Department of Zoology, College of Basic Science and Humanities, Orissa University of Agriculture and Technology, Bhubaneswar, 751003 India; 4 Redox Biology Laboratory, Department of Zoology, Center of Excellence in Environment and Public Health, Ravenshaw University, Cuttack, 753003 Odisha India; 5 Central Research Laboratory, IMS and Sum Hospital, Siksha ‘O’ Anusandhan University, K-8 Kalinga Nagar, Bhubaneswar, 751003 Odisha India; 6 National Bureau of Agriculturally Important Microorganisms, Indian Council of Agricultural Research, Mau, Uttar Pradesh 275103 India; 7 Medical Biotechnology, Department of Biochemistry, Pt. J.N.M. Medical College, Raipur, C.G. 49200 India; 8 Department of Biotechnology, Utkal University, Bhubaneswar, 751004 India; 9 Present Address: ICMR-Regional Medical Research Centre (ICMR-RMRC), Chandrasekharpur, Bhubaneswar, Odisha 751023 India; 10 Present Address: Department of Bioinformatics, SRM University Delhi-NCR, Sonepat, Haryana 131029 India

**Keywords:** Biomechanics, Molecular medicine

## Abstract

Oxidative stress is implicated in both hypo- and hyper-thyroid conditions. In the present study an attempt has been made to elucidate possible interaction between vitamin E or/and curcumin (two established antioxidants) with active portion (redox signaling intervening region) of nuclear factor erythroid 2-related factor 2 (NRF2) as a mechanism to alleviate oxidative stress in rat heart under altered thyroid states. Fifty Wistar strain rats were divided into two clusters (Cluster A: hypothyroidism; Cluster B: hyperthyroidism). The hypo- (0.05% (w/v) propylthiouracil in drinking water) and hyper- (0.0012% (w/v) T4 in drinking water) thyroid rats in both clusters were supplemented orally with antioxidants (vitamin E or/and curcumin) for 30 days. Interactive least count difference and principal component analyses indicated increase in lipid peroxidation, reduced glutathione level, alteration in the activities and protein expression of antioxidant enzymes like superoxide dismutase, catalase, glutathione peroxidase and glutathione reductase under altered thyroid states. However, the expression of stress survival molecules; nuclear factor κB (NFκB) and the serine-threonine kinase B (Akt), in hyper-thyroidism only points towards different mechanisms responsible for either condition. Co-administration of vitamin E and curcumin showed better result in attenuating expression of mammalian target for rapamycin (mTOR), restoration of total protein content and biological activity of Ca^2+^ ATPase in hyperthyroid rats, whereas, their individual treatment showed partial restoration. Since NRF2 is responsible for activation of antioxidant response element and subsequent expression of antioxidant enzymes, possible interactions of both vitamin E or/and curcumin with the antioxidant enzymes, NRF2 and its regulator Kelch ECH associating protein (KEAP1) were studied *in silico*. For the first time, a modeled active portion of the zipped protein NRF2 indicated its interaction with both vitamin E and curcumin. Further, curcumin and vitamin E complex showed *in silico* interaction with KEAP1. Reduction of oxidative stress by curcumin and/or vitamin E may be due to modulation of NRF2 and KEAP1 function in rat heart under altered thyroid states.

## Introduction

Thyroid hormones (THs) exert significant actions on energy metabolism. Thyroid hormones modulate cellular oxidative stress (OS) through mitochondrial oxygen consumption *in vivo*. Altered THs level causes subtle changes in the ratio of antioxidant enzymes leading to imbalance in clearance of reactive oxygen species (ROS). Excess ROS accumulation causes deteriorative effects to proteins, lipids and DNA present in their vicinity^1^. The basal level of ROS is maintained by active oxygen scavenging through enzymatic superoxide dismutase (SOD), catalase (CAT), glutathione peroxidise (GPx) and glutathione reductase (GR) and non-enzymatic antioxidant molecules like the reduced glutathione (GSH)^2^.

Several genes like myosin isoform, calcium cycling proteins and protein kinases that encode important structural and regulatory proteins in the myocardium, are TH responsive, thereby regulate cardiac performance^1^. Hyper-thyroidsm is associated with tachycardia, increased protein concentration, mitochondrial respiration, ROS generation and DNA damage in rat heart^3^. Hypo-thyroidism, on the other hand, is considered generally a hypometabolic state; nevertheless, generation of ROS is not proportionately lower in this dysfunction in comparison to euthyroid individuals^4^. The ubiquitously expressed transcription factor, nuclear factor κB (NFκB) and the serine-threonine kinase (protein kinase B, PKB; AKT), are activated in response to stress and involved in controlling the balance between survival and apoptosis in cells^5^. Cardiac hypertrophy is an adaptive response in case of increased volume and pressure overload and there is a positive correlation between OS and cardiac hypertrophy in experimental hyper-thyroidism^6^. It has been reported that the AKT-mammalian target for rapamycin (mTOR) signalling pathway is activated in the hypertrophied hearts of hyper-thyroid animals^7^. In recent years, considerable interest is being shown towards the use of antioxidant molecules as therapeutic agents to target OS induced pathophysiologic disorders^8^. Vitamin E (VIT-E) and curcumin (CRM) are low molecular mass natural antioxidants. Vitamin E is a widely studied molecule and several authors have proposed that VIT-E supplementation could be useful in attenuation of OS mediated pathophysiology^9^. Curcumin is a biologically active phenolic compound derived from the rhizome of Indian spice turmeric (*Curcuma longa*) and used in traditional medicine. It possess potent antioxidant, anti-inflammatory and anti-carcinogenic properties^10^.

Nuclear Factor erythroid-derived 2-like 2 (NRF2) is the master regulator of antioxidant response via induction of Antioxidant Response Element (ARE) for expression of antioxidant enzymes. However, NRF2 is primarily regulated by Kelch-like ECH-associated protein 1 (KEAP1). In the development of therapeutic and preventive medications for an array of diseases and disorders, the Keap1–Nrf2 protein–protein interaction (PPI) has emerged as a target for up regulation of the ARE-controlled cyto-protective oxidative stress response enzymes. These agents are mostly electrophilic molecules that act by modifying the sulfhydryl groups of cysteine residues on KEAP1 and inhibit Keap1–Nrf2 PPI; thereby, activate NRF2/ARE. Nevertheless, it is hypothesized that these electrophilic indirect inhibitors may have “off-target” side effects via interaction with cysteine residues of other vital cellular proteins^11^. Therefore, there is a need to understand the mechanism by which extraneous electrophilic compounds exert their effects on NRF2-KEAP1 PPI.

With this background, the present investigation was carried out to study the cardio-protective role of individual or co-administration of VIT-E and CRM against OS and energy sensing (Ca^2+^ ATPase) under hypo- and hyper-thyroid conditions along with elucidation of the signaling mechanisms. One of the important hypotheses was whether the signaling of the antioxidant enzymes by NRF2 was mediated by VIT-E/CRM or not (Supplementary Fig. 1). Therefore, in the present investigation, through *in silico* analysis we predict the direct interaction of the VIT-E and CRM with the transcription factor nuclear factor erythroid 2–related factor 2 (NRF2), which is responsible for antioxidant response. The results were further validated through study of OS index (LPx, lipid peroxidation) and antioxidant response, NFkB-mediated redox signaling and Ca^2+^ signaling as an index of cardiac contractility to have an in depth understanding of the efficacy of extraneous antioxidant supplementation (VIT-E and CRM) in altered thyroid status induced cardiac dysfunction.

## Materials and Methods

### Chemicals

L-thyroxine (T4), 6-propyl-2-thiouracil (PTU), CRM, Triton-X-100 and thiobarbituric acid were purchased from Sigma Chemical Co., USA. L–methionine, hydroxylamine hydrochloride, ethyelenediamine tetra acetic acid, riboflavin, phenol red, orthophosphoric acid, Sulfanilamide, N-(l-naphthyl) ethylenediaminedihydrochloride, hydrogen peroxide (H_2_O_2_), sodium hydroxide, ascorbic acid, ferric chloride, trichloroacetic acid, butylatedhydroxytoluene, metaphosphoric acid, and sodium dodecyl sulfate were obtained from SISCO Research Laboratories, Mumbai, India. All other chemicals otherwise not mentioned were of analytical grade.

### Animal model and experimental design

The study was conducted under the guidance of Institutional Animal Ethics committee (IAEC) as approved by Committee for the purpose of supervision and experimentation on animals (CPCSEA), Government of India. Fifty adult male Wistar rats aged 150 ± 10 days obtained from the National Institute of Nutrition (Hyderabad, India) were used in the present study. The animals were housed in the animal room, maintained at 25 ± 2 °C with 12 h artificial illuminations followed by12 h darkness. They were given water and food *ad libitum* during the study and were fasted over night before sacrifice. Rats were divided into two separate clusters. In hypo-thyroid animal model (Cluster-A), the rats were allocated randomly into five groups, consisting of five animals each. Group IA was control while Groups -IIA, IIIA. IVA and VA were rendered hypo-thyroid by administering 0.05% 6-propyl-thiouracil (PTU) in their drinking water for 30 days. Similarly in the hyper-thyroid animal model (Cluster-B), the rats were allocated randomly into five groups of five animals each. Group IB was control while Groups IIB, IIIB. IVB and VB were rendered hyper-thyroid by administering 0.0012% thyroxine (T_4_) in their drinking water for 30 days. The Groups- IIIA and IIIB were treated with VIT-E (200 mg kg^−1^ body weight) while Groups-IVA and IVB were treated with CRM (30 mg kg^−1^ body weight) for 30 days. On the other hand, Groups-VA and VB received both VIT-E and CRM. Olive oil was used as vehicle. Rats of Groups I (A, B) and II (A, B) received same amount of olive oil daily for the entire study period. The doses of PTU, T4, VIT-E and CRM were taken from previous published study of our group^12,13^.

### Tissue processing for biochemical estimations

Frozen heart tissue were taken out from −80 °C and 20% (w/v) homogenate (with the help of Potter-Elvejhem type, motor driven glass Teflon homogenizer at 250 rpm speed with 7–8 up and down strokes at 4 °C) was prepared in 50 mM phosphate buffer (pH 7.4) with 1 mM PMSF as anti-protease (referred as crude homogenate). The crude homogenates were centrifuged at 12, 000 × g for 10 min at 4 °C in a cooling centrifuge (Model C-24, REMI, Mumbai, India) to sediment nuclei and tissue debris. Aliquots of the crude homogenate of all samples were subjected to centrifugation at 600 × g for 10 min and 4 °C and the pellet (cell debris and nuclear fraction) was discarded. The supernatant was then centrifuged at 10,000 × g for 20 min at 4 °C to pellet the mitochondrial fraction and the post-mitochondrial fraction (PMF) was used for assay of all enzymes and proteins except Ca^2+^ ATPase activity which was measured in the mitochondrial pellet fraction (membrane component). For assay of SOD, GPx and GR, the PMF was passed through Sephadex G-25 prior to enzyme assay to get rid of low molecular weight interfering compounds. All parameters were assayed as described below.

### Assay of LPx

LPx was assayed in the crude homogenate according to the method of Ohkawa *et al*. following the formation of thiobarbituric acid reactive substances (TBARS) whose concentration was calculated from its extinction coefficient 1.56 × 10^5^ M^−1^ cm^−1^ and expressed as nmol TBARS formed mg^−1^ protein^14^.

### Assay of antioxidant enzyme activities

The PMFs obtained as mentioned above were used for the assay of antioxidant enzymes. The SOD assay was carried out according to the modified nitrite method of Das *et al*.^15^, which involves generation of superoxide radical by photoreduction of riboflavin and its detection by nitrite formation from hydroxylamine hydrochloride at 543 nm using Greiss reagent. The CAT activity was assayed according to the method Aebi^16^ following the decrease in absorbance of hydrogen peroxide at 240 nm after blocking compound-I formation according to Cohen *et al*.^17^. Enzyme activity was calculated taking 43.6 M^−1^ cm^−1^ as molar extinction coefficient of H_2_O_2_ and expressed as µKatal mg^−1^ protein. The GPx activity was assayed by measuring rate of NADPH oxidation using tert-butylhydroperoxide and GSH as substrates in presence of extraneous GR^18^. The GR activity was assayed by measuring rate of NADPH oxidation in using the oxidized glutathione (GSSG) as substrate^19^. Activities of both the enzymes were calculated from the molar extinction coefficient of NADPH (6.22 × 10^3^ M^−1^ cm^−1^) and expressed as nmoles of NADPH oxidized^−1^ min^−1^ mg^−1^ protein.

### Assay of Ca^2+^ ATPase activity

The activity of Ca^2+^ ATPase was assayed in the membrane fraction (10,000 × g pellet of the crude homogenate dissolved in the homogenizing buffer) according to the method of Roy and Chainy^20^. The samples (40 µg protein) were incubated in standard incubation medium consisting of 50 mM Tris-HCl, pH 7.4, 0.5 mM EGTA and 5 mM Ca^2+^. After pre-incubation of 5 minutes at 37 °C, the reaction was started by addition of 0.1 ml of 3 mM ATP and the final reaction volume was made up to 1 ml with Tris-HCl buffer and incubated for 30 minutes at 37 °C in a shaking water bath. The reaction was stopped by addition of 0.1 ml of ice cold 50% (w/v) Trichloroacetic acid (final conc. 5%). Control determinations were made by omitting the enzyme, Ca^2+^ or the ATP. This assay mixture was then centrifuged at 5000 rpm for 10 minutes and the liberated inorganic phosphate in the supernatant was measured according to the method of Chen *et al*.^21^. Enzyme activity was expressed as µmol inorganic phosphate released h^−1^ mg^−1^ protein.

### Assay of non-enzymatic antioxidant molecule GSH

The reduced glutathione content was estimated in the metaphosphoric acid treated deproteinized supernatant of the crude homogenate using GSH as standard^22^.

### Estimation of protein content

The protein content was estimated with the help of Bradford reagent^23^, using bovine serum albumin as standard.

### Western blotting

The heart tissue was homogenized in 50 mM Tris-HCl buffer, pH 7.4, containing 150 mm NaCl, 104 mM PMSF, 100 μM E-64, 80 μM aprotinin, 100 μM leupeptin, 1% Triton-X-100 and 0.1% SDS to avoid protein degradation. Homogenates were centrifuged at 1000 × g for 20 min at 4 °C. After resolving in 12% SDS-PAGE the proteins were transferred to 0.4 μm PVDF membrane (Pall Life Sciences) at 22 mA current for 1 h. The membrane was blocked in blocking solution (5% w/v skimmed milk) for 1 h, followed by treatment with primary rabbit polyclonal antibodies (anti-SOD1, anti-CAT, anti-pAKT, anti-tAKT, anti-mTOR and anti-NFĸB p65 and anti-G3PDH; Imgenex India Pvt. Ltd., Bhubaneswar; and anti-SOD2; Santa Cruz, Biotechnology, USA) at room temperature with constant rocking. The membrane was washed three times with washing solution and subsequently incubated with HRP-conjugated anti-rabbit goat IgG at room temperature. After washing, specific immuno-reactive proteins were detected with ECL kit of Santa Cruz in X-ray film and their expression level was measured by densitometry (Image-Quant TL, Image Analysis Software version 2003)^24^.

### *In silico* analyses

The targeted amino acid sequences such as, SOD1, SOD2, ATP2b4, ATPase, CAT, GPx, AKT, mTOR and NRF2 were retrieved from the UniProt KB data base (http://www.uniprot.org/) for homology modeling. Based on suitable correlation with the target sequence, sequence identity and query coverage, a suitable template for each target sequence was selected through BLASTp tool. After selection of suitable template, SWISS-MODEL was used for homology modeling. The homology models for targets, mTOR was not meant for modeling due to unavailability of suitable template. Ramachandran plot was used for validation of generated homology models before undertaking docking study using the tool, RAMPAGE. Ligands, VIT-E and CRM were retrieved from PubChem data base for molecular docking by AutoDockVina software, as reported previously. Discovery studio visualizer and LigPlus were used for target-ligand interactions. For the first time, we predicted a valid *in silico* structure (of the active target site) of NRF2 using comparative modeling approach. The detailed protocols for docking of the CRM and VIT-E with respective proteins may be found elsewhere (Supplementary Text)^25^.

Autodock-4.2 software was used to study the interaction of CRM ligand (CID: 969516) and VIT-E (14985) with the NRF2 receptor separately as well as collectively. The molecular docking of selected ligand-compound against modeled 3D structure of NRF2 protein was performed using ADT GUI of Autodock v4.2. Ligand preparation involves addition of hydrogen atoms and Gasteiger charges. The number of active torsions on CRM and VIT-E ligands were found to be 10 and 13, respectively. Receptor preparation was done by making whole macromolecule-centered 3D grid of 126 × 126 × 126 with spacing of 0.553 Å. The conformational search of the ligand was performed by applying the Lamarckian genetic algorithm. The other docking parameters selected were: 10 GA runs, population size of 150, maximum energy evaluations in the range of 250,000, maximum number of generations 27,000, mutation rate 0.02, cross-over rate 0.8 and all other parameters are set to the default values of the software. ADT GUI was used for visualization of our docking results. The detailed protocols for *in silico* approaches may be found elsewhere^26–29^.

### Statistical analyses

All sets of data were subjected to test for homogeneity of variance and normal distribution. Data were presented as mean ± standard deviation. Statistically significant differences between the treatment means were calculated by subjecting the data to one-way analysis of variance (ANOVA) followed by Least Significant Difference (LSD) test. Minimal statistical significance was accepted at p < 0.05. Principal component analysis (PCA) is a mathematical procedure wherein a number of (possibly) correlated variables are transformed into a (smaller) number of uncorrelated variables called principal components. In this technique, the first principal component accounts for as much of the variability in the data as possible while each succeeding component accounts for as much of the remaining variability as possible. Statistical analysis software version 9.0 was used to evaluate LSD and PCA Factor analyses.

## Results

### Total protein content

The total protein content significantly (p < 0.04) decreased in the hearts of all hypo-thyroid rats with or without antioxidant treatment (Table 1). On the contrary, the total protein content was elevated (p < 0.04) in the hyper-thyroid rats and this elevation was restored partially by CRM treatment and completely in co-treatment groups (Table 2).

**Table 1:** Effect of vitamin E (VIT-E) or/and curcumin (CRM) on the studied biochemical parameters in heart of hypothyroid rats.

	Control	PTU	PTU + VIT-E	PTU + CRM	PTU + VIT-E + CRM
Protein content	17.60 ± 2.30^*^	11.40 ± 1.20^$^	13.20 ± 0.17^$^	14.30 ± 0.15^$^	13.2 ± 0.21^$^
LPx	3.08 ± 0.21^*^	4.10 ± 0.35^$^	2.66 ± 0.27^*^	2.68 ± 0.31^*^	2.64 ± 0.36^*^
Ca^2+^ ATPase	0.72 ± 0.06^*^	0.62 ± 0.04	0.65 ± 0.03	0.61 ± 0.06	0.64 ± 0.08
GSH	0.74 ± 0.06^*^	0.64 ± 0.04^$^	0.72 ± 0.04^*^	0.75 ± 0.06^*^	0.71 ± 0.03^*,$^
SOD	13.50 ± 0.96^*^	11.62 ± 0.58^$^	11.92 ± 0.80^$^	14.66 ± 1.20^*^	11.46 ± 0.50^$^
CAT	0.21 ± 0.01^*^	0.18 ± 0.02^$^	0.192 ± 0.01^$^	0.21 ± 0.02^*,$^	0.20 ± 0.02^*,$^
GPx	0.34 ± 0.01^*^	0.29 ± 0.02^$^	0.29 ± 0.02^$^	0.29 ± 0.02^$^	0.32 ± 0.01^*^

Protein content (µg g^−1^ tissue wet weight), LPx (nmol TBAR mg^−1^ protein), Ca^2+^ ATPase (nmol Pi^−1^ hr^−1^ mg^−1^ protein), GSH (µmolesg^−1^ tissue) and activities of SOD (units mg^−1^ protein), CAT (µ Katal mg^−1^ protein) and GPx (nmoles of NADPH oxidized min^−1^ mg^−1^ protein) in the rat heart under altered thyroid states. Data are expressed as mean ± S. D. of three independent observations carried out in triplicate. Data having different superscript symbols such as * and ^$^ indicate significant with each other at p < 0.05.

**Table 2:** Effect of vitamin E or/and curcumin on the studied biochemical parameters in heart of hyperthyroid rats.

	Control	PTU	PTU + Vit E	PTU + Cur	PTU + Vit E + Cur
Protein content	16.20 ± 1.50^*^	21.40 ± 2.50^$^	20.30 ± 1.90^$^	19.50 ± 1.90^*,$^	19.20 ± 2.00^*^
LPx	3.58 ± 0.52^*^	5.12 ± 0.50^$^	3.37 ± 0.40^*^	3.26 ± 0.41^*^	3.78 ± 0.23^*^
Ca^2+^ ATPase	0.74 ± 0.09^*^	0.96 ± 0.09^$^	0.65 ± 0.13^*^	0.62 ± 0.11^*^	0.65 ± 0.12^*^
GSH	0.69 ± 0.05^*^	0.53 ± 0.07^$^	0.70 ± 0.10^*^	0.72 ± 0.09^*^	0.79 ± 0.10^*^
SOD	12.60 ± 1.14^*^	16.80 ± 1.13^$^	13.96 ± 1.40^*^	14.06 ± 1.40^*^	12.24 ± 1.00^*^
CAT	0.20 ± 0.02^*^	0.16 ± 0.01^$^	0.23 ± 0.03^*^	0.20 ± 0.02^*^	0.25 ± 0.01^*^
GPx	0.34 ± 0.02^*^	0.27 ± 0.03^$^	0.30 ± 0.01^$^	0.32 ± 0.02^*,$^	0.35 ± 0.02^*^

Protein content (µg g^−1^ tissue wet weight), LPx (nmol TBARS mg^−1^ protein), Ca^2+^ ATPase (nmol Pi^−1^ hr^−1^ mg^−1^ protein), GSH (µmolesg^−1^ tissue) and activities of SOD (units mg^−1^ protein), CAT(µ Katal^−1^ mg^−1^ protein) and GPx (nmoles of NADPH oxidized min^−1^ mg^−1^ protein) in the rat heart under altered thyroid states. Data are expressed as mean ± S. D. of three independent observations carried out in triplicate. Data having different superscript symbols such as * and ^$^ indicate significant with each other at p < 0.05.

### Ca^2+^ ATPase activity

The activity of Ca^2+^ ATPase was significantly (p ≤ 0.03) increased in the hyper-thyroid group (Gr IIA) in comparison to respective control rats (Table 2). Oral administration of the antioxidant compounds both singly and in combination reduced the activity to the basal level. However, no significant change (p > 0.07) was observed in any of the hypo-thyroid groups (Gr IB-VB) either with or without antioxidant treatment (Table 1).

### LPx level

Endogenous LPx level in the crude homogenate of heart was significantly increased (p < 0.05) in both the altered thyroid groups (Gr IIA, IIB) in the absence of any antioxidant supplementation. However, treatment with VIT-E and/or CRM prevented the hypo- and hyper-thyroid rats from damage due to LPx (Tables 1 and 2).

### Active oxygen scavenging enzyme activities and protein expression

The biological activity of cardiac SOD decreased (p < 0.05) in the hypo-thyroid rats and increased in hyper-thyroid condition in the absence of any antioxidant supplementation. CRM alone significantly restored the activity of SOD in both the altered thyroid conditions, however, VIT-E failed to normalize SOD activity in the hypo-thyroid state. The activity of CAT was reduced (p < 0.05) in both the altered thyroid groups (Gr II A, B) which reverted back partially in hypo-thyroid and completely in hyper-thyroid rats when administered with antioxidant molecules. The GPx activity declined (p ≤ 0.04) in both the rat models, and the decline was more prominent in hyper-thyroid group in the absence of any antioxidant supplementation. VIT-E and CRM treatment failed to restore GPx activity in the hypo-thyroid condition. On the other hand, in the hyper-thyroid state, individual treatment of antioxidants partially augmented GPx activity, whereas their co-treatment completely restored GPx activity to the level of control (Tables 1 and 2).

**Figure 1:**
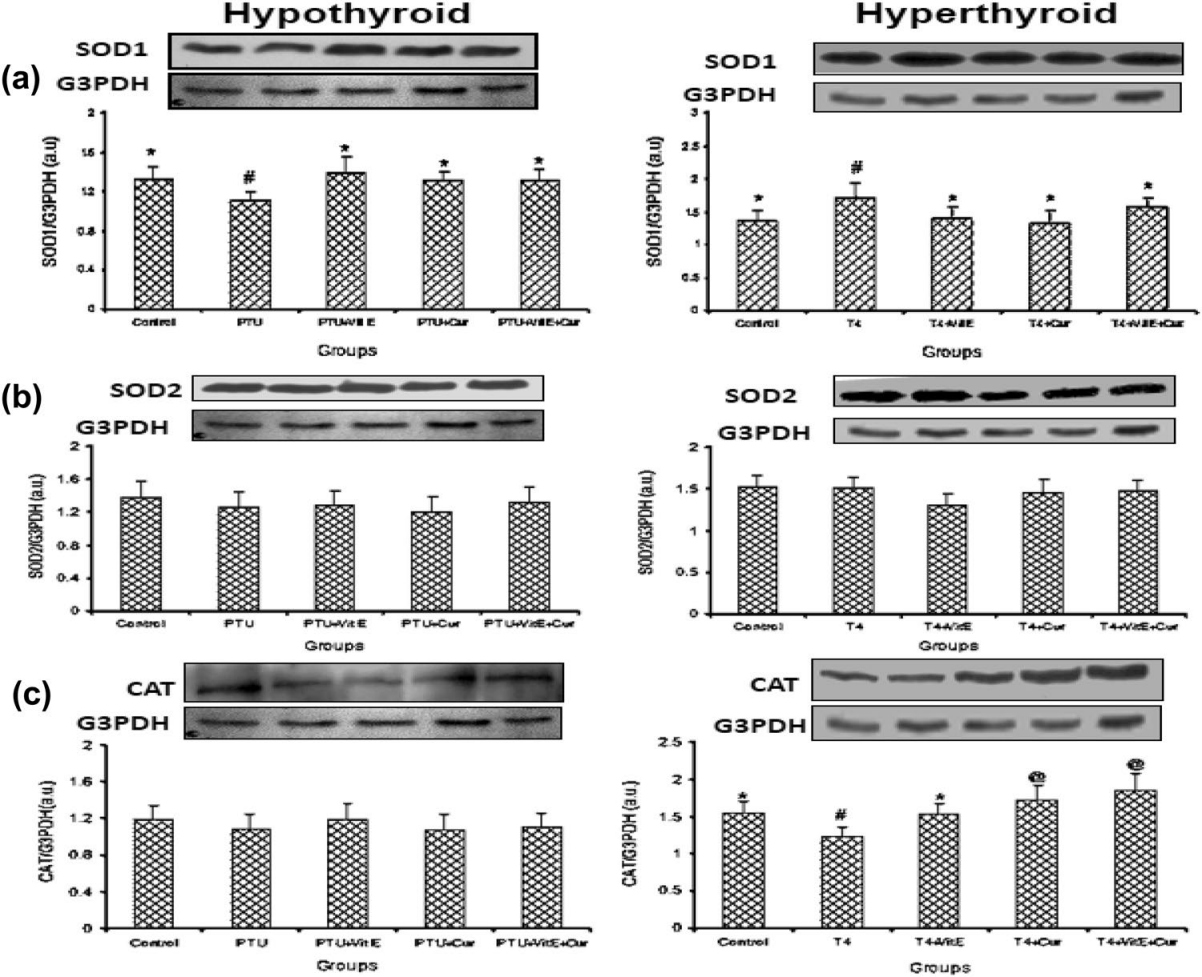
Effects of vitamin E and/or curcumin on expression of antioxidant enzymes (SOD and CAT) in rat heart under altered thyroid states (**a**) SOD1; (**b**) SOD2 and (**c**) CAT protein expressionrepresented as western blot image and corresponding densitogram. Data are expressed as mean ± S.D. of three independent observations carried out in triplicate. Data having different superscript are significant at p < 0.05.

More or less similar results were obtained when the translated products of active oxygen scavenging enzymes were monitored by Western blotting. Densitometry revealed that SOD1 (Cu-Zn-SOD) expression decreased (p < 0.05) in the PTU-treated and increased (p < 0.05) in the T_4_-treated rats in the absence of any antioxidant supplementation. Nevertheless, VIT-E and/or CRM brought back the altered protein expression of SOD1 to that of control in both the altered thyroid states (Fig. 1a,b). There was no change in the protein expression of SOD2 in either of the altered thyroid conditions (Fig. 1c,d). The CAT expression decreased (p < 0.05) in the T_4_ treated rats whereas, administration of antioxidant molecules (VIT-E and CRM) increased (p < 0.05) CAT expression above the basal level. However, this increase was more highlighted in the CRM (Gr IVB) and combined antioxidant administration (Gr VIIIB). There was no change in the protein expression of CAT in the hypo-thyroid groups with or without antioxidant supplementation with respect to control (Fig. 1e,f).

### GSH Content

The GSH content decreased (p ≤ 0.03) in both the thyroid abnormalities, although more prominently in hyper-thyroid rats and was reverted back to basal level in all the antioxidant molecules supplemented groups in both the experimental models (Tables 1 and 2).

### Expression of AKT and downstream molecules

**Figure 2:**
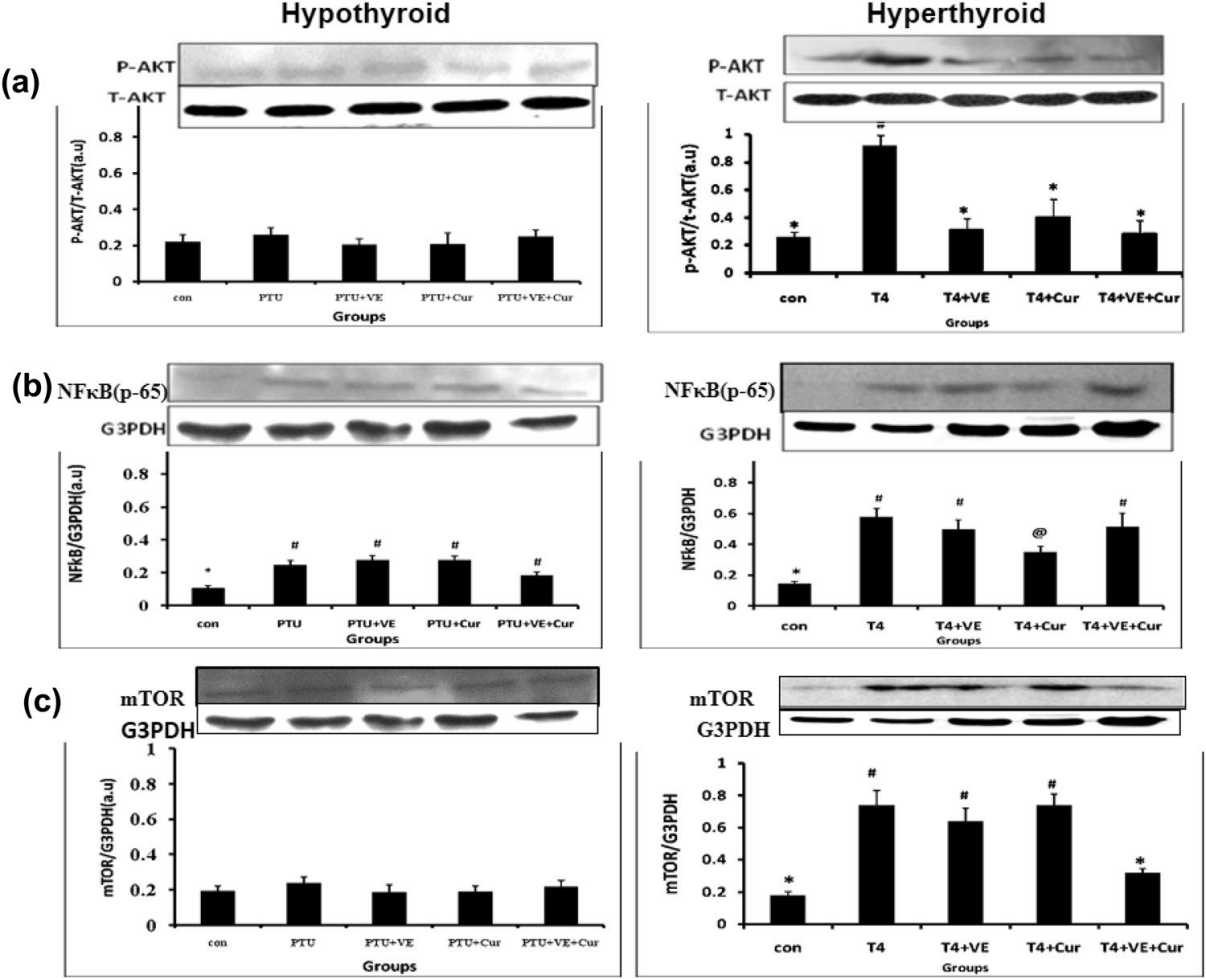
Effects of vitamin E and/or curcumin on expression of stress responsive cell survival pathway in rat heart under altered thyroid states (**a**) pAKT; (**b**) NFκB and (**c**) mTOR protein expressionrepresented as western blot image and corresponding densitogram. Data are expressed as mean ± S.D. of three independent observations carried out in triplicate. Data having different superscript are significant at p < 0.05.

A significant (p < 0.05) augmentation in the level of p-AKT, mTOR, and NFκB (p65) was noticed in the T_4_ treated rats in the absence of antioxidant supplementation. Administration of VIT-E and/or CRM showed differential result with respect to their effect on level of p-AKT and their downstream molecules. Individual administration of CRM down regulated AKT phosphorylation (p-AKT) and NFκB levels (Fig. 2a–d). VIT-E administration reduced the level of p-AKT. However, their co- treatment successfully reduced the levels of p-AKT and mTOR (Fig. 2a,f) to basal level. On the other hand, in the hypo-thyroid hearts antioxidant treatments failed to elicit any response in the profile of these stress response signaling proteins (Fig. 2a–f).

### Results from *in silico* studies

Molecular docking scores (kcal/mol) of VIT-E (as ligand-1: L1) andCRM (as ligand-2: L2) against individual target proteins are presented in Table 3. Individually, VIT-E had effective docking score of −9.4 kcal mol^−1^ and −8.36 kcal mol^−1^, respectively, against GPx and ATPase. Similarly, CRM had effective docking score of −5.71 kcal mol^−1^ against GPx. Therefore, both VIT-E (L1) and CRM (L2) may be having an interactive pathway with GPx. Similarly, the target-ligand interactions for all other studied proteins were also studies. Ala 89 in SOD1 was found to interact with both the ligands while Asn 97 was found to interact with L2 only. In SOD2, Gln 215 and Lys 154 were found to firmly dock with L1 and L2, respectively. For GPx, Ser 205 was shown to interact with L1 while both Arg 200 and Lys 268 were found to dock with L2. Thr 361 of ATPase had shown a good docking with L2. The ligands were not suitable for interaction with CAT, p-AKT and mTOR due to their large size and the presence of steric hindrance by amino acids in the targeted protein structures that gave low docking score (data not given).

**Table 3:** Docking score for the redox regulatory and other studied enzymes/proteins.

Target enzyme	Docking score (kcal/mol)	
	Ligand-1 (vitamin-E)	Ligand-1 (curcumin)
SOD1	−4.55	−5.11
SOD2	−4.75	−3.94
CAT	ND	ND
ATPase	−8.36	−4.90
GPx	−9.40	−5.71
p-Akt	ND	ND
Nrf2*	SS	SS
mTOR	ND	ND

ND- not done (was not suitable for docking). *SS-separately modeled and studied.

### Modeling results of NRF2

**Figure 3:**
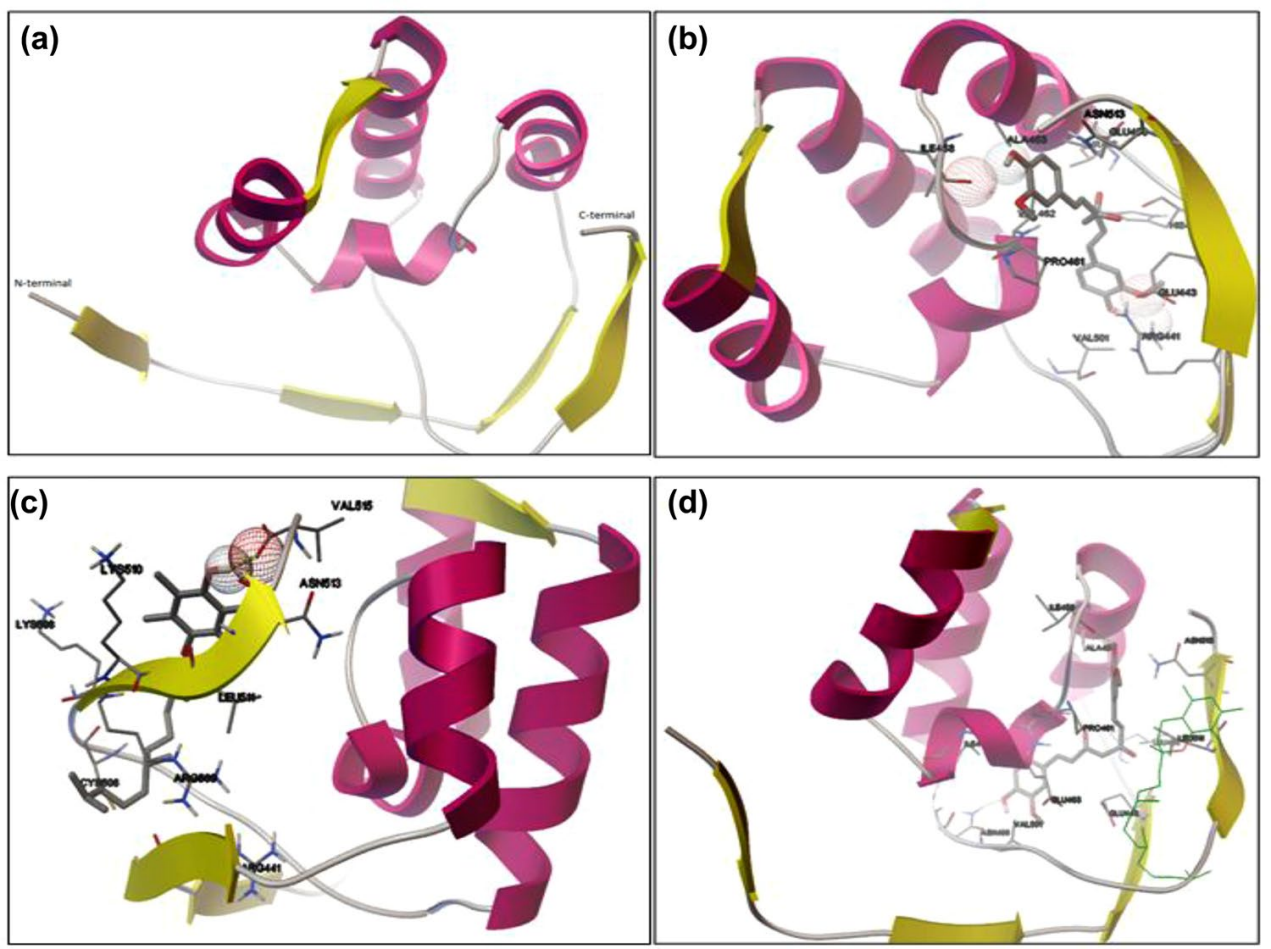
*In silico* binding of VIT-E/RCM with NRF2. (**a**) The predicted *in silico* stricture of NRF2. (**b**) Docked conformation of Curcumin ligand at the binding site of NRF2 receptor. (**c**) Molecular docking model of Vitamin E ligand with NRF2 receptor. (**d**) Showing binding mode and interactions of curcumin ligand with the docked complex of Vitamin E-Nrf2 complex. Curcumin ligand is shown in stick style and coloring scheme is atom type. Vitamin E is in line style in green color. The interacting amino acids in the receptor are shown in line style, and rest of the amino acid residues in secondary structure representation.

NRF2 is an important signaling molecule that mediate cellular ROS metabolism. However, 3D- structure (crystal/*in silico*) of NRF2 is not available in the structural databases. Its redox sensing IVR region is important for ROS metabolism (Supplementary Fig. 1). The downloaded sequence of NRF2 consists of 597 amino acids. Therefore, for the sake of studying the structural and functional properties, it structure was modeled. The BLAST algorithm was used for searching highly similar template sequences whose 3D structures were available in databases. The amino acid residues present at the active portion were conserved while the conservation of the whole sequence was very low. There is a general understanding in structural biology that during evolution, the amino acids present in the active portion were highly conserved because they are important for protein function. Therefore, despite having less sequence identity, we decided to predict the 3D structure of its active portion on the basis of different template selection using Modeller 9v7. Sequences that gave high sequence identity with the template and high model score was selected for further analyses (Table 4). This software implements comparative protein modeling by satisfaction of spatial restraints. Of the three predicted structures from comparative modeling, the structure having highest sequence identity (90%) with the template region (2–90) of the protein (PDB ID: 2IZ1A) with an e-value of zero was chosen. The NRF2 protein structure is visualized using PyMol software. The predicted 3D active region of NRF2 protein consists of four alpha helices that are interconnected by loop regions (Fig. 3a). Then the ligands L1 and L2 were docked with the predicted structure.

**Table 4:** Structure prediction using different template selection for NRF2.

Target	Modeled segment of target	Template PDB code	Template PDB segment	Sequence identity	E-value	Model score
NP_113977.1 597 aa	427–515	2IZ1A	2–90	90.00	0.00	1.00
	460–552	4EOTA	227–317	25.00	0.11	0.89
	494–543	2DGCA	230–270	32.00	0.00	0.03

NRF2 is an important redox sensing molecule albeit no data were present to have its in silico studies with any ligands due to its zipped nature that lead to very low template score. For the first time, the most active site was predicted as mentioned in the table and interacted with the ligands.

### Interaction of CRM and VIT-E with the modeled NRF2 protein

The docked complex of Curcumin Ligand (CID: 969516) binds at the main active-site of NRF2 receptor with the lowest binding energy of −7.98 kcal. The possibilities of binding of the ligand to some other binding sites were also detected, but the minimum energy cluster shows highest binding affinity for the main active-site. The interaction between curcumin and NRF2 protein involves Arg441 and Ile458 amino acids through hydrogen bonding network (Fig. 3b). The nearby close contact residues are Glu443, His445, Thr447, Glu450, Ala453, Val501, Asn513, Pro461 and Val462.

**Figure 4:**
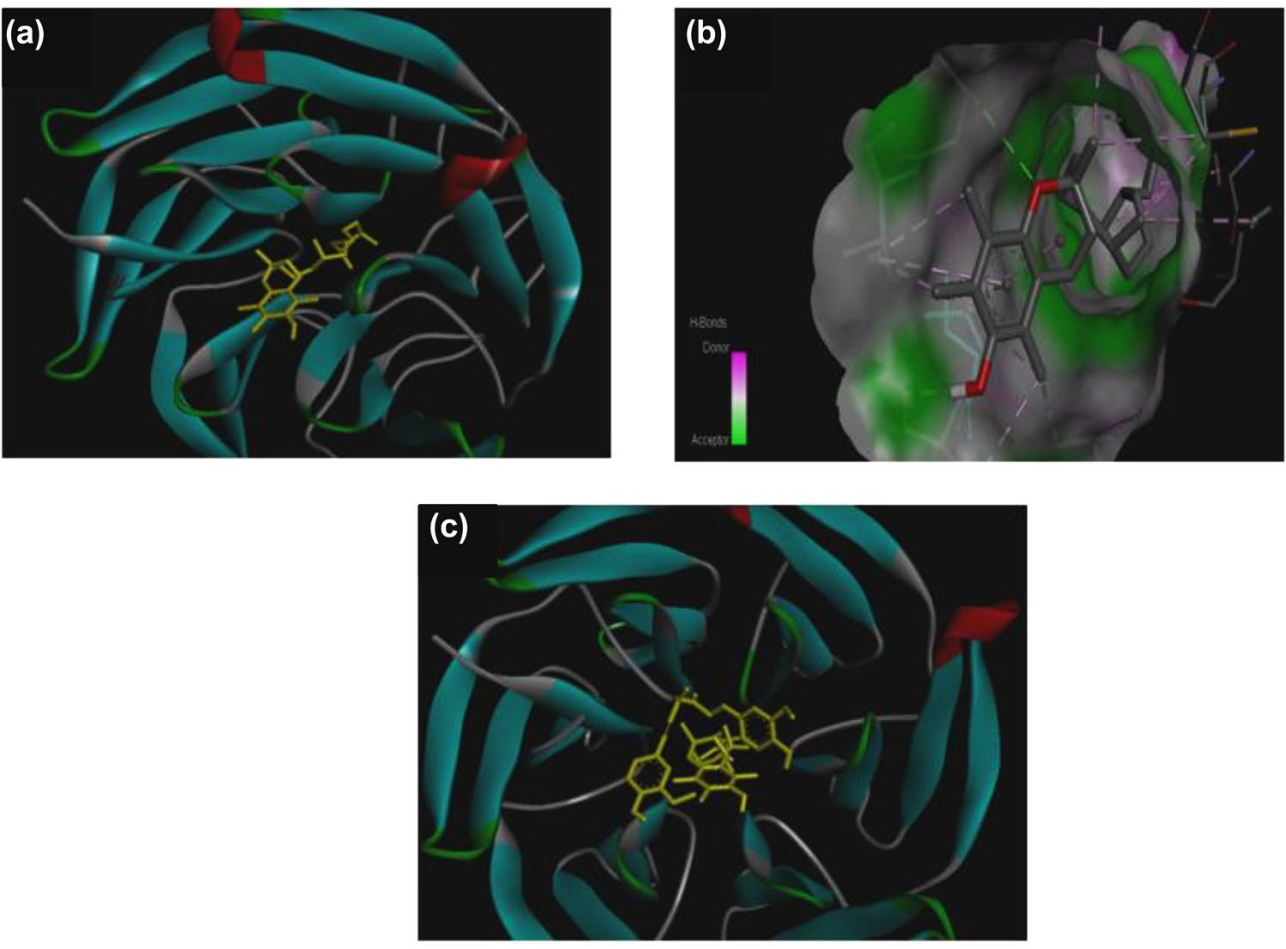
Docking of KEAP1 with (**a**) VIT-E, (**b**) CRM and (**c**) both VIT-E and CRM.

The lowest energy cluster of VIT-E ligand (CID:14985) binds at the active portion of NRF2 protein with a binding energy of −5.94 Kcal. The ligand interacts at the active portion of NRF2 protein with the hydrogen bonds, involving Val515 (Fig. 3c). The close-contact residues that are responsible for holding the ligand at the binding pocket are Arg441, Cyst506, Lys508, Arg509, Lys510, Leu511 and Asn513. Later on, the docked complex of VIT-E-NRF2 was considered as a receptor protein, and docking simulations were performed against CRM ligand. The resultant complex showed 12 conformers in the lowest energy cluster with a mean binding energy of −7.86 kcal, and its lowest binding energy was −8.27 Kcal. CRM ligand was shown to be attached with the active portion of the receptor complex with the hydrogen bonded network, involving Ile458 and Asn499 amino acids (Fig. 3d). The nearby residues involve in non-bonding interactions were Glu443, Leu446, Ala453, Asn499, Val501, Leu511, Asn513, Val462, Glu463 and Ile466. Similarly, KEAP1 (Kelch-like ECH-associated protein 1) crystal structure was taken from PubMed. Docked with VIT-E (Fig. 4a)/CRM (Fig. 4b) alone with KEAP1 showed a docking score of −6.73 kcal mol^−1^. When both the antioxidants were taken together (Fig. 4c) the docking score was −10.35 kcal mol^−1^.

### LSD and PCA analyses

**Figure 5:**
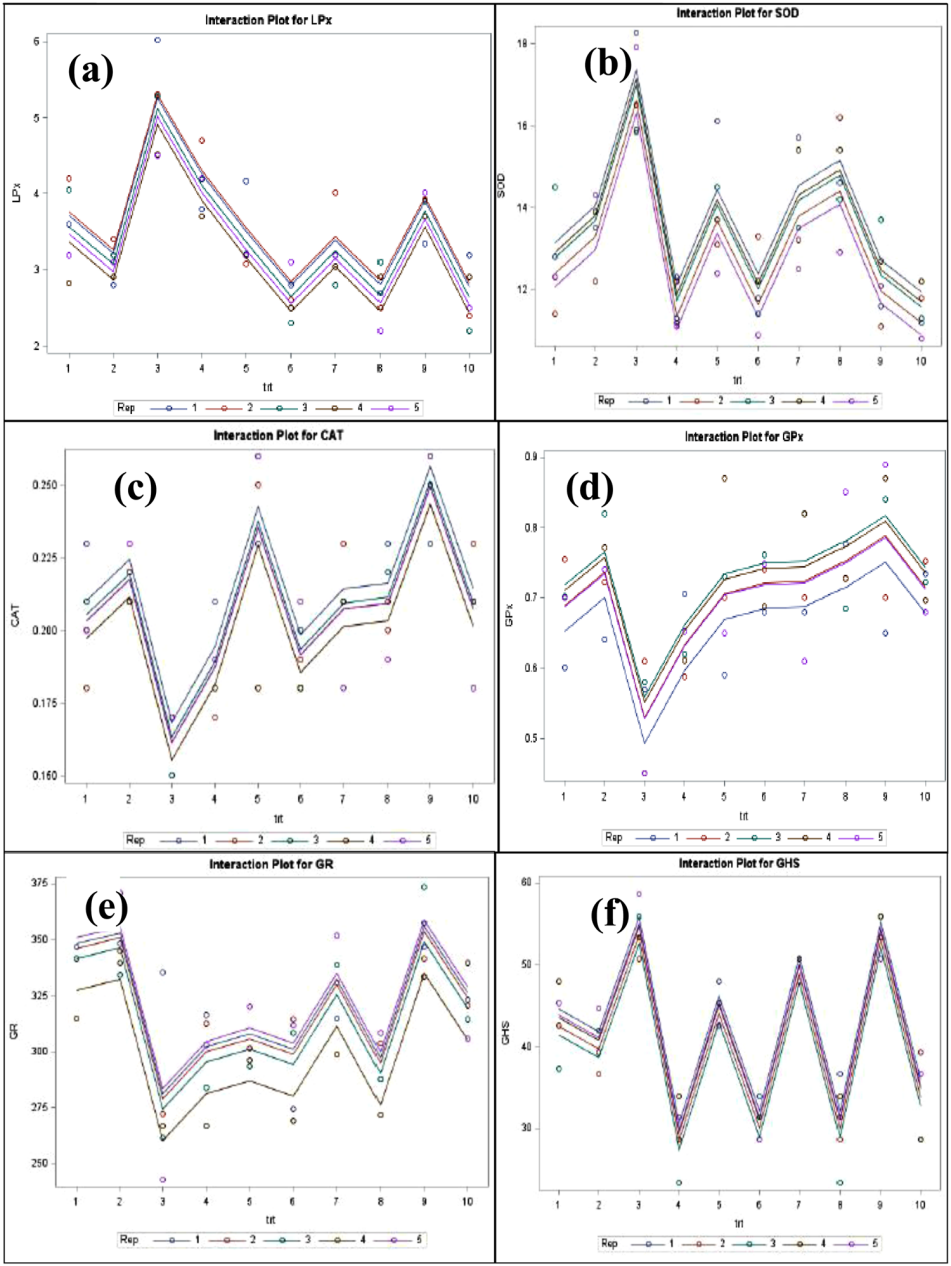
Interactive plot for LSD for individual parameters. Self descriptive interactive plot for (**a**) lipid peroxidation, (**b**) superoxide dismutase, (**c**) catalase, (**d**) glutathione peroxidase, (**e**) glutathione reductase, (**f**) reduced glutathione.

**Figure 6:**
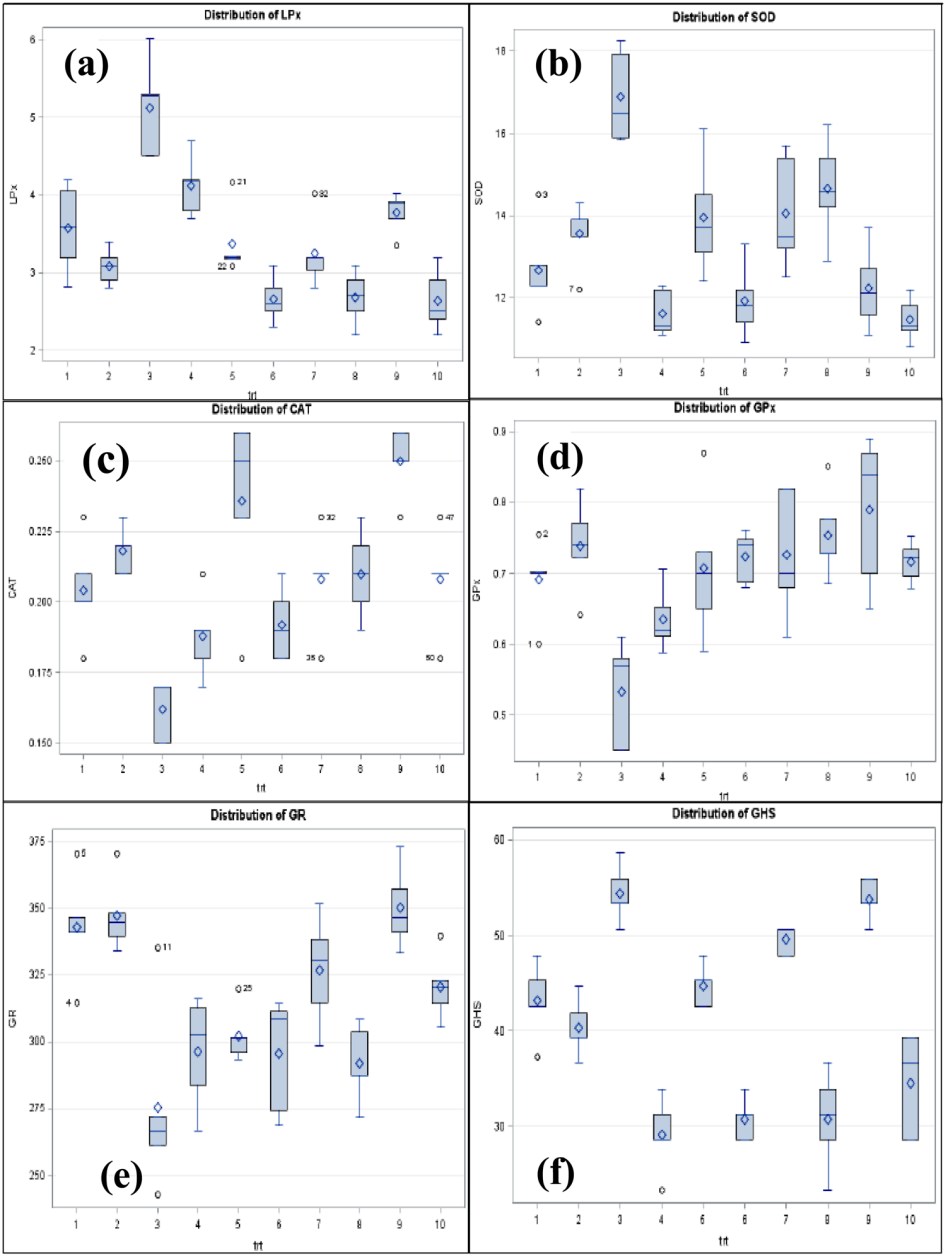
Distribution plot of studied each parameter. Distribution plot for (**a**) lipid peroxidation, (**b**) superoxide dismutase, (**c**) catalase, (**d**) glutathione peroxidase, (**e**) glutathione reductase, (**f**) reduced glutathione.

For post-hoc comparison of two treatment means LSD was performed after ANOVA while to study the association among the groups, PCA was done. The results are presented in Figs 5 and 6 and Supplementary Table 1. Both the distribution plot (Fig. 5) and interactive plot indicate that LPx, (Figs 5a and 6a), SOD (Figs 5b and 6b), CAT (Figs 5c and 6c), GPx (Figs 5d and 6d), GR (Figs 5e and 6e) and GSH (Figs 5f and 6f) are quite well influenced by CRM/VIT-E treatment in rat heart tissues as the distribution was highly distinct for each parameter.

**Figure 7:**
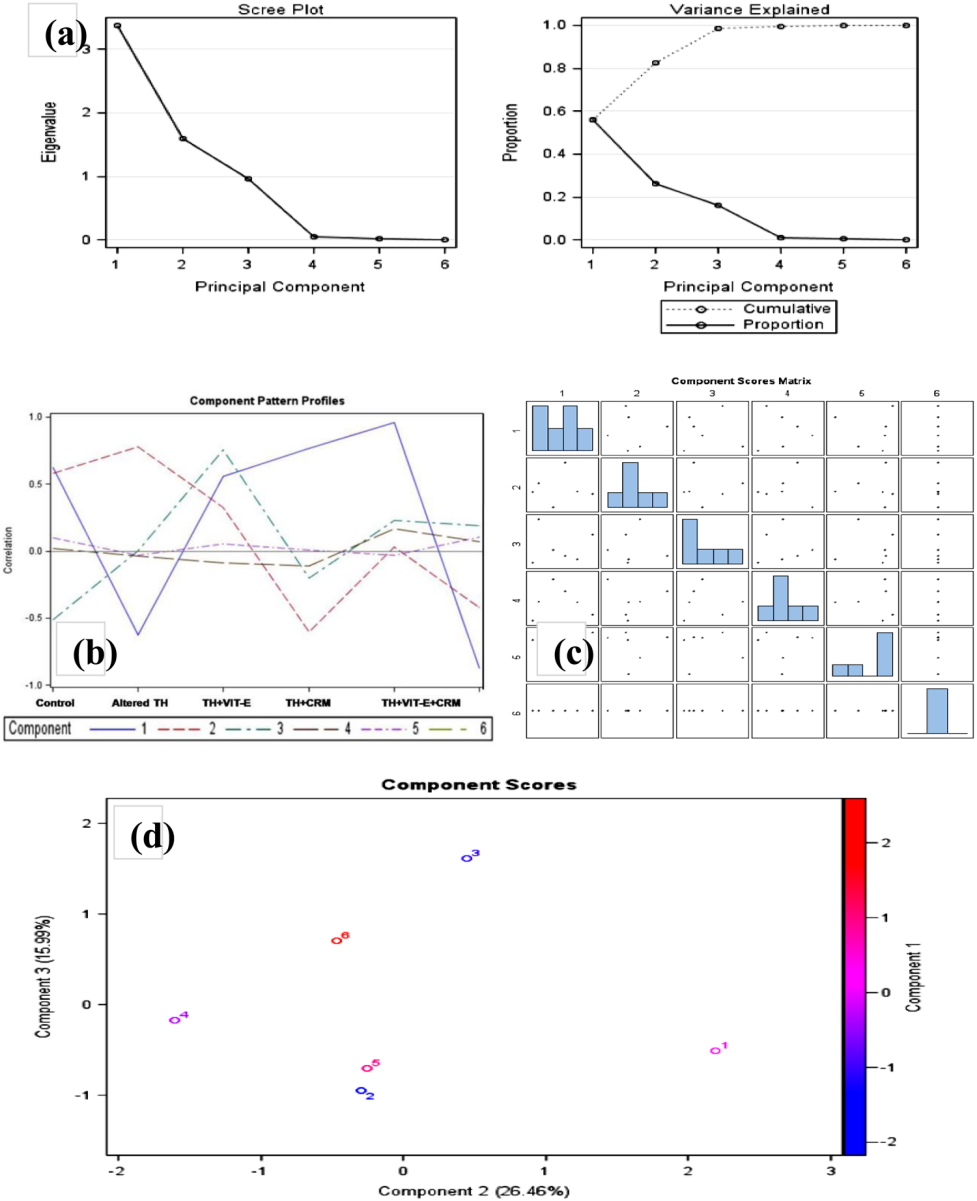
Principal component pattern analysis. (**a**) Screw plot analysis showing the interaction of parameters. (**b**) Component pattern profile; (**c**) component scored matrix and (**d**) Component scores for the six parameters 1: LPx, 2: SOD, 3: CAT, 4: GPx, 5: GR, and 6: GSH are very distinctly observed.

The fact is further supported by the scree plot (Fig. 7a). The scree test is a test for determining the number of factors to retain in a factor analysis or principal components analysis. A Scree Plot is a simple line segment plot that shows the fraction of total variance in the data as represented by each principal component (PC). The PCs are ordered, and by definition are therefore assigned a number label, by decreasing order of contribution to total variance. One can use the size of the eigen value to determine the number of principal components. Using the Kaiser criterion, the PC with the largest eigen values (>1) were retained (PC1 and PC2), which explained cumulative and proportion variance within 0.0 to approximately 0.6 (Fig. 7b) and a clear distribution of component pattern profile in either side of correlation with values mostly >0.5 (Fig. 7a). The higher the proportion, the more variability that the principal component explains. The size of the proportion helps in deciding whether the principal component is important enough to retain. Here, the principal component 1 with a proportion of >0.5 explains >50% of the variability in the data. Therefore, this component is important to include. Most importantly a clear variation within the groups for each parameter was revealed by component pattern profile (Fig. 7b) and the component scored matrix (Fig. 7c) with a component score within −2 to +3. Therefore, each treatment group had a variation from one another, mostly the V^th^ group with combined CRM and VIT-E administration showing a recovery from OS in comparison to the individual VIT-E or CRM administrated groups under both the altered thyroid conditions

## Discussion

The present investigation is a comparative study of cardiac OS under hypo- and hyper-thyroid state in rat model to understand the efficacy and mechanism of extraneous antioxidant supplementation combining wet lab and *in silico* experiments.

### Effects of VIT-E and CRM on Ca^2+^-ATPase and ROS metabolism

Calcium ions are essential for cardiac contractions and relaxation. ROS act as cardio depressant through impairment of Ca^2+^ homeostasis. As TH regulates the sequestration of intracellular Ca^2+^ into sarcoplasmic reticulum via Ca^2+^-ATPase, it is natural to have an augmented Ca^2+^-ATPase activity in hyper-thyroid animals which is in agreement with earlier reports^30,31^. However, no change in Ca^2+^-ATPase activity was reported in hypo-thyroid rats. The T_4_-induced ROS causes intracellular Ca^2+^ influx mediated by increased membrane LPx. Lipid peroxidation is a major index of OS and an increase in its level in the present study indicates oxidative damage in both the altered thyroid conditions. Thyroid hormone is principally associated with acceleration of basal metabolic rate and oxidative metabolism, therefore, hyper-thyroidism leads to higher oxygen consumption and increased generation of ROS resulting in elevated LPx in rat heart^3^. On the other hand, hypo-thyroidism is characterized by reduced oxidative metabolism and markedly increased lipid and lipoprotein levels in the plasma which is reported to be more susceptible to ROS attack with a consequent elevation of lipid peroxide^32^. In fact, it is reported that mitochondria of mammalian cells produce superoxide radicals under hypoxic condition leading to induction of OS similar to that of hypo-thyroid state where cellular respiration is low^33^. Our findings on LPx in both the hypo- as well as hyper-thyroid heart tissues are in concordance with the above reports. This increase in LPx in both the thyroid abnormalities is associated with decrease in the level of the small tripeptide, non-enzymatic antioxidant molecule GSH which is widely involved in maintaining the cellular redox status and also serves as a substrate for GPx and GST-conjugation reaction^2^. Decreased level of GSH could be a consequence of its decreased synthesis or increased consumption or may be due to its role in neutralization of the elevated ROS level which is evident from increased LPx level in the tissue. This is in good agreement with earlier report of Mogulkoc *et al*.^33^.

VIT-E and curcumin are potent antioxidant molecules with effective ROS scavenging activity, however, their mode of action is different. VIT-E is a lipophilic antioxidant molecule which when incorporates into the lipid bilayer interferes with the formation of lipid peroxides and carbonyl groups via its scavenging action on alkyl, alkoxyl, and peroxyl radicals resulting in attenuated LPx and protein oxidation, respectively. It prevents LPx damage in the tissue by trapping the chain propagating peroxyl radicals and thereby reduces the length of the auto-oxidation chain^34,35^. It also serves as a powerful scavenger of ^·^OH and acts directly with a variety of ROS such as singlet oxygen, LPx products and ${{\rm{O}}}_{2}^{\cdot -}$ to form relatively harmless tocopheryl radical^36^. On the other hand, CRM with its unique conjugated structure which includes two methoxylated phenols and an enol form of β-diketone shows radical-trapping activity. The antioxidant mechanism of CRM may include any one of the following interactions, namely, scavenging or neutralizing free radicals, ${{\rm{O}}}_{2}^{\cdot -}$ quenching and making it less available for oxidative reactions or interacting with oxidative cascade and preventing its outcome^37,38^. Several studies suggest that CRM inhibits oxidation of low density lipoprotein (LDL) which plays an important role in the development of atherosclerosis, one of the major causes of heart failure^39^. Thus, it is speculated that antioxidant molecules VIT-E and/or CRM efficiently reduced the LPx level by quenching the ROS in hyper-thyroid state and reducing hyper-lipidemia in hypo-thyroid state. Conformational change in Ca^2+^-ATPase is caused by CRM, thereby blocks the ATPase from binding ATP and inhibits Ca^2+^ entry into microsomes and sarcoplasmic reticulum SR^40^. It has been reported that VIT-E down regulates SR Ca^2+^ ATPase when administered to rabbits after one week of myocardial infarction^41^. Our finding corroborates the above observations. By virtue of its free radical scavenging action, VIT-E would reduce the production of carbon-centered radicals capable of reacting directly with biomolecules, such as -SH groups of proteins resulting in the reduction of consumption and exhaustion of GSH^42^. Addition of GSH to one of the two α, β-unsaturated C-C double bonds of CRMcatalyzed by GST is one of the mechanisms for removing CRM from cells. This removal of CRM-conjugated GSH enhances cells with increased biosynthesis mechanisms for replenishing the lost GSH. In addition, CRM increases the cellular GSH levels by enhancing the transcription of glutamate-cysteine ligase (Gcl), the rate limiting enzyme in GSH synthesis^43^. Supplementation of VIT-E and CRM augmented the level of GSH to that of control under hypo- and hyper-thyroidism.

### Effects of VIT-E and CRM on antioxidant defense

Oxidative stress causes overproduction of pro-oxidants and as a result destabilizes antioxidant enzymes. The altered antioxidant enzyme activity as well as their expression level in the present study also gives evidence in support of induction of OS in response to PTU as well as T_4_ treatment (hypo- and hyper-thyroid state). In ROS mediated oxidative damage SOD acts as the first line of defense by catalyzing the dismutation of superoxide anion (${{\rm{O}}}_{2}^{\cdot -}$) to H_2_O_2_ which is turn is detoxified by CAT and GPx^2^. Regulation of all the studied antioxidant enzymes was affected by the level of TH. Hyper-thyroidism upregulated the activities of SOD and GR while the activities of CAT and GPx were down regulated. On the other hand, hypo-thyroidism decreases the activity as well as expression of all the studied redox regulatory enzymes. Hyper-thyroidism increases oxygen consumption and produces ${{\rm{O}}}_{2}^{\cdot -}$ due to leakage in mitochondrial electron transport chain^3^. The increased activity as well as expression of SOD in the hyper-thyroid rat model may be a cellular adaptive response to neutralize the toxic effect of ${{\rm{O}}}_{2}^{\cdot -}$. This result is in corroboration with earlier report which suggests that over expression of antioxidant network, namely SOD1 and SOD3 in mice protects against ROS-mediated LPx^44^. On the other hand, the decreased activity of SOD in hypo-thyroid rat hearts might be due to the decrease in the SOD1 expression and unaltered SOD2 expression which is in good agreement with the results of Pasupathi and Latha^45^. Conversion of H_2_O_2_ the toxic hydroxyl radical is prevented by its metabolism in H_2_O by CAT and GPx^2^. The role of CAT in antioxidant defense depends on the levels of its expression and the cellular concentrations of H_2_O_2_. We found a reduced CAT activity in both the altered thyroid states. The decline in activity of CAT in the hyper-thyroid rats may be due to modification of transcriptional factors responsible for the initiation of transcription^46,47^. The rat CAT gene sequence includes binding sites for TH as well as for various transcription factors like NFκB^47^. It has been reported that ROS induces the activation of redox-sensitive transcription factors like NFκB which upon translocation into the nucleus possibly suppress CAT expression after binding to its promoter region^46^. Therefore, declined CAT activity and expression in hyper-thyroid condition as found in the present study may be due to overexpression of NFkB in response to TH. On the other hand, decrease in activity of CAT in hypo-thyroid model may be attributed to decrease of its substrate, H_2_O, which may be due to decrease activity of SOD enzyme. The major protective role of GPx, may reside in the fact that it is the only antioxidantenzyme that metabolizes three major ROS like H_2_O_2_, lipid peroxide (LOOH) and peroxynitrite (ONOO^·−^)^2^. GPx along with GR plays an important role in balancing the redox status of the tissue. It uses GSH as the substrate during catalysis of H_2_O_2_ to H_2_O and in due course the reduced glutathione (GSH) is converted to the oxidized glutathione (GSSG). In return GSSG is recycled to GSH by the enzyme GR. The decreased GPx activity in both hypo- as well as hyper-thyroid rats may be due to the damage incurred to the enzyme by LPx products which are significantly augmented in PTU and T_4_ treated rats probably by a modification of the selenocysteine residue at the active site of the enzyme^48^. Studies indicate that GPx is more susceptible to oxyradical inactivation compared to SOD and CAT^49^. Inactivated GPx by free radicals has been reported to be degraded by proteases thus reducing their intracellular levels as seen in the present rat models under both the altered thyroid states^50^. Hypo-thyroidism causes a reduced metabolic state which is reflected in decreased activity of all studied antioxidant enzymes and a decrease in total protein content of the tissue. The results of the present study supplements the fact that a perturbed antioxidant defense, an augmented Ca^2+^-ATPase activity (in hyper-thyroidism), an elevated LPx as well as reduced GSH level are responsible for OS induction in altered thyroid states.

A differential regulation of both biological activity and protein expression of major antioxidant enzymes were observed in response to VIT-E and/or CRM under hypo- and hyper-thyroid state. While CRM treatment alone ameliorated the altered activities of SOD and CAT in both the altered thyroid conditions, VIT-E alone could stabilize SOD and CAT in hyper-thyroid model only and failed to elicit any response in the hypo-thyroid model. Moreover, their combined administration successfully normalized GPx and GR activities as compared to their individual administration. The results are in good agreement with previous reports^12,24,51^. Therefore, to understand the mechanism of their regulation, the expression profile of upstream regulators AKT, mTOR and NFĸB were studied.

### Effects of VIT-E and CRM on AKT- NFĸB signaling in hyper-thyroid state

In hyper-thyroid condition, the alteration of the antioxidant enzymes especially the augmentation of total SOD activity and SOD1 protein expression, indicate towards the possibility of its modulation through distinct regulatory pathways that are directly or indirectly modulated by cellular redox status. We focused on the cell survival pathway, proposing T_4_-induced ROS (${{\rm{O}}}_{2}^{\cdot -}$) as the initiator candidate, since it was previously demonstrated that ROS may lead to AKT phosphorylation^6^. Activated AKT then phosphorylates IĸB kinase which in turn, causes activation and nuclear translocation of NFĸB–dependent pro-survival genes^52^. Our study demonstrated a causal relationship between activation of AKT and induction of Cu-Zn-SOD (SOD1), suggesting the role of T_4_ in induction of SOD1 expression via activation of AKT and NFĸB as a mechanism of protection of cardiac cells against OS. Therefore, the down-regulation of pAKT by administration of VIT-E and/or CRMrestored the activities and expression of SOD1 in hyper-thyroid groups. However, both the antioxidant molecules failed to restore the protein expression of CAT. On the other hand, hypo-thyroid condition is essentially a hypoxic condition with decreased mitochondrial oxidative phosphorylation and less production of ${{\rm{O}}}_{2}^{\cdot -}$ ^33^. Thus, as SOD1 is down-regulated in absence of its substrate (${{\rm{O}}}_{2}^{\cdot -}$), expression of pAKT as well as NFĸB (p65) is not detected in hypo-thyroidism. Therefore, the enhanced ROS generation in hypo-thyroidism may be attributed to hyper-lipidemia induced LPx. Therefore, it is proposed that the cause as well as mechanism of ROS generation is different in both the altered thyroid conditions.

### Effects of VIT-E and CRM on AKT- mTOR pathway in hyper-thyroid state

Earlier reports have proposed that TH-induced hypertrophy is phenotypically similar to the physiological hypertrophy mediated by AKT signaling, suggesting overlapping mechanism^53^. Kuzman et al. have shown that T_4_ activates mTOR (a master regulator of protein synthesis) and p70S6k signaling^6^. As increased protein synthesis is an important criteria for hypertrophy, activation of mTOR might be an important pathway through which TH induce cardiac hypertrophy. To corroborate the findings, in the present study we observed an augmentation in mTOR expression together with increased protein content in hyper-thyroid rat heart. We speculate that administration of both VIT-E and CRM cooperatively deactivated the mTOR pathway whereas either of them individually is not capable of doing so. This result is in corroboration with the result of Subudhi et al., where they showed a decrease in heart weight with respect to body weight (HW/BW) in response to T_4_ treatment (under same experimental conditions), and this alteration was nearly restored in the combined antioxidant treatment groups^13^. On the other hand, the expression of mTOR was not detected in any of the hypo-thyroid groups which coincide with the decrease protein content. This outcome is in support with earlier report where an unaltered HW/BW was found in hypo-thyroid rats with or without supplementation of antioxidants^12^.

### Possible role of VIT-E and CRM on NRF2 and KEAP1 activation

Molecular docking scores indicate that VIT-E had effective docking score against GPx and ATPase. Similarly, CRM had effective docking score against GPx. Similarly, SOD1 and SOD2 were found to interact with both the ligands. Therefore, VIT-E and CRMmay have possible interactive pathway with SOD, GPx and ATPase for redox regulation and energy homeostasis. The individual docking score for both VIT-E and CRM for NRF2 and docked complex of VIT-E-NRF2 for CRM ligand indicate a strong possibility of interaction of NRF2 with both the antioxidant small molecules. The resultant complex shows 12 conformers in the lowest energy cluster with a mean binding energy of −7.86 kcal, and its lowest binding energy was −8.27 Kcal. CRM ligand attached to the active region of the complex receptor with the hydrogen bonded network, involving Ile458 and Asn499 amino acids. The nearby residues such as Glu443, Leu446, Ala453, Asn499, Val501, Leu511, Asn513, Val462, Glu463 and Ile466 showed non-bonding interactions signifying the possibility of the hypothesis that NRF2 may be activated in rat heart under VIT-E/CRM administration. The three component transcription pathway which include antioxidant response element (ARE), NRF2 and KEAP1 are the principal regulators of antioxidant gene expression. The steady state level of NRF2 is maintained by KEAP1 as its association drives NRF2 to proteolytic degradation. Removal of KEAP1 due to Cys modification by small molecules is reported to activate NRF2^11^. NRF2 activation in VIT-E/CRM treatment may be attributed to the excellent *in silico* interaction of these two extraneous antioxidants with KEAP1^54,55^. Since the interactions of VIT-E/CRM do not include Cys residues of KEAP1^11^, it may be suggested that their administration will not have “off-target” side effects via interaction with Cys residues of other cellular proteins.

**Figure 8:**
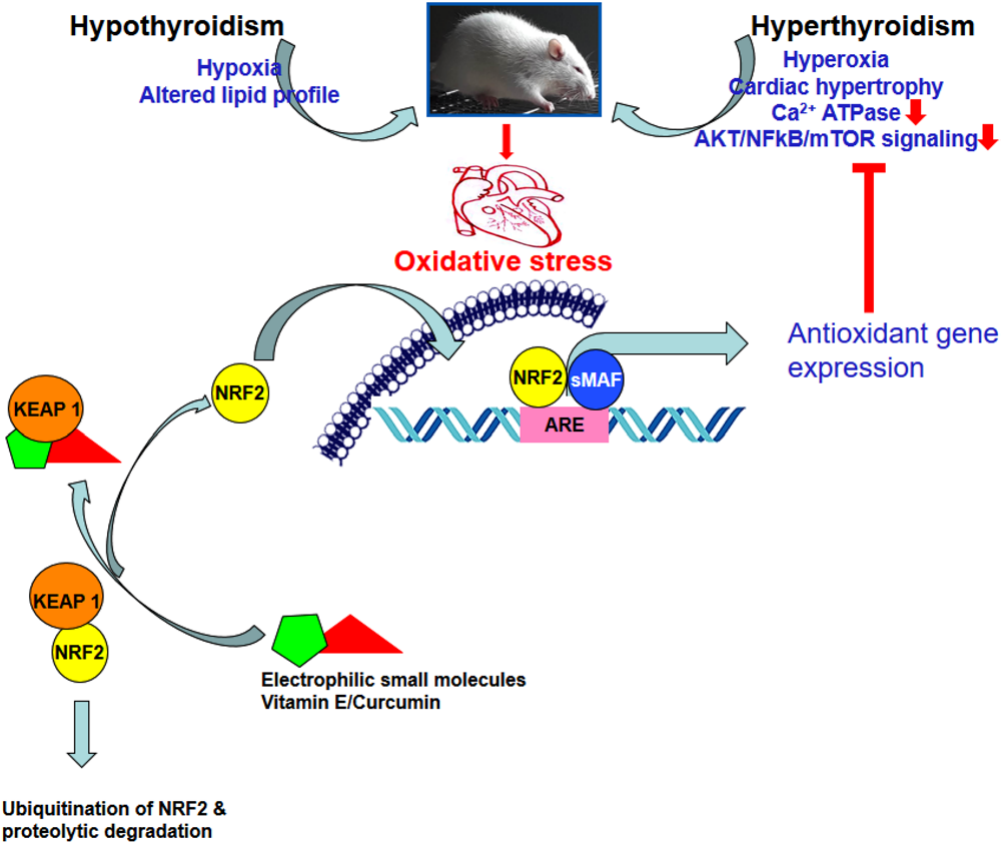
Schematic representation for the regulation of antioxidant genes by NRF2-KEAP1 interaction via electrophilic small molecules such as Vitamin E and Curcumin under altered thyroid condition in rat heart. Antioxidant gene expression could repress Ca^2+^ ATPase and AKT/NFkB/mTOR signaling under hyperthyroid state. Administration of vitamin E and curcumin collectively could better work to neutralize altered thyroid hormone induced oxidative stress in rat heart.

The present study points out the protective role of VIT-E and CRM on mitigation of OS and restoration of antioxidative potential in cardiac tissue under altered thyroid condition. The cause of ROS generation as well as the mechanism(s) of amelioration of OS parameters was dissimilar in both the altered thyroid conditions as evidenced by activation ofcell survival pathway (NFĸB, AKT, andmTOR signalling) in hyper-thyroidism only. In addition, the mechanism(s) of mitigation of oxidative damage by individual or co- administration of VIT-E and CRM was different in hypo- and hyper-thyroid rats. Different combinations of antioxidant molecules would work better against OS and newer combinations may be tried along with routine therapy to protect the heart from oxidative injury. Based on both wet laboratory and *in silico* results, it may be hypothesized that VIT-E and CRM inhibit LPx either by way of direct scavenging of ROS or via increase in enzymatic antioxidant defence through KEAP1 binding thereby interfering with NRF2-KEAP1 protein-protein interaction leading to ARE activation (Fig. 8). However, the later proposed mechanism needs laboratory approaches for confirmation. Role of Ca^2+^ ATPase as energy sensors, mTOR and AKT as kinases and NFkB as cell survival factor in stabilisation of the redox regulation under VIT-E and/or CRM administration in hyper-thyroid states may be useful in mitigating cardiac damage under altered thyroid states in general and reducing the risk of hyperthyroidism induced heart failure or stroke in particular.

## Supplementary information

Supplementary Information
