# Supplementary material for: Possible activation of NRF2 by Vitamin E/Curcumin against altered thyroid hormone induced oxidative stress via NFĸB/AKT/mTOR/KEAP1 signalling in rat heart
Source: Sci Rep. 2019 May 15;9:7408. doi: 10.1038/s41598-019-43320-5 (PMC6520394; doi:10.1038/s41598-019-43320-5)
Supplement: Supplementary file 1 — Supplementary Information [file 41598_2019_43320_MOESM1_ESM.pdf]

Possible activation of NRF2 by Vitamin E/Curcumin against altered thyroid hormone induced oxidative stress via NFκB/AKT/mTOR/KEAP1 signalling in rat heart

Pallavi Mishra[1,2,†], Biswaranjan Paital[1,3,*,†], Srikanta Jena[4], Shasank S. Swain[5], Sunil Kumar[6], Manoj K Yadav[7], Gagan BN Chainy[8], Luna Samanta[4,*]

[1]Department of Zoology, Utkal University, Bhubaneswar-751004, India

[2]Department of Zoology, Government Autonomous College, Phulbani, Kandhamal, Odisha

[3]Redox Regulation Laboratory, Department of Zoology, College of Basic Science and Humanities, Orissa University of Agriculture and Technology, Bhubaneswar-751003, India

[4]Redox Biology Laboratory, Department of Zoology, Center of Excellence in Environment and Public Health, Ravenshaw University, Cuttack-753003, Odisha, India

[5]Central Research Laboratory, IMS and Sum Hospital, Siksha 'O' Anusandhan University, K-8 Kalinga Nagar, Bhubaneswar 751003, Odisha, India.

[6]National Bureau of Agriculturally Important Microorganisms, Indian Council of Agricultural Research, Mau (Uttar Pradesh) 275103, India

[7]Medical Biotechnology, Department of Biochemistry, Pt. J.N.M. Medical College, Raipur (C.G.)-49200, India

[8]Department of Biotechnology, Utkal University, Bhubaneswar-751004, India

***Corresponding authors:**

Biswaranjan Paital, Redox Regulation Laboratory, Department of Zoology, College of Basic Science and Humanities, Orissa University of Agriculture and Technology, Bhubaneswar-751003, India

Email: biswaranjanpaital@gmail.com, Tel: +91-674-2397964, Fax: +91-674-2397780

and

Luna Samanta**,** Redox Biology Laboratory**,** Department of Zoology, Center of Excellence in Environment and Public Health, Ravenshaw University, Cuttack-753003, Odisha

Email: lsamanta@ravenshawuniversity.ac.in; luna_samanta@rediffmail.com, Telefax: +91-671-2200160

[†]both have equal contribution.

Materials and methods for Modeling and docking studies

The whole sequence of NRF2 did not give a suitable template identity score based on the query coverage (was < 12 % for NRF2 whereas >30 % is required for modeling). Since, interaction between VIT-E/CRM and NRF2 was one of the most important objectives of this study, the most active portion of NRF2 was predicted and was docked with VIT-E/CRM individually or together. To the best of our knowledge, the three dimensional *in silico*/crystal structure of NRF2 is not available. For the first time, we predicted a valid *in silico* structure of NRF2 using comparative modeling approach. The protein sequence of NRF2 (NP_113977) of *Rattus norvegicus* (Norway rat) was downloaded from NCBI database. BLAST algorithm was used to find similarity in sequence as well as structure against the structural database. Protein structure prediction via modeler produced a set of alternative models from which the best and most accurate model has been selected.

Supplementary Fig. 1 Different sites in Nrf 2.

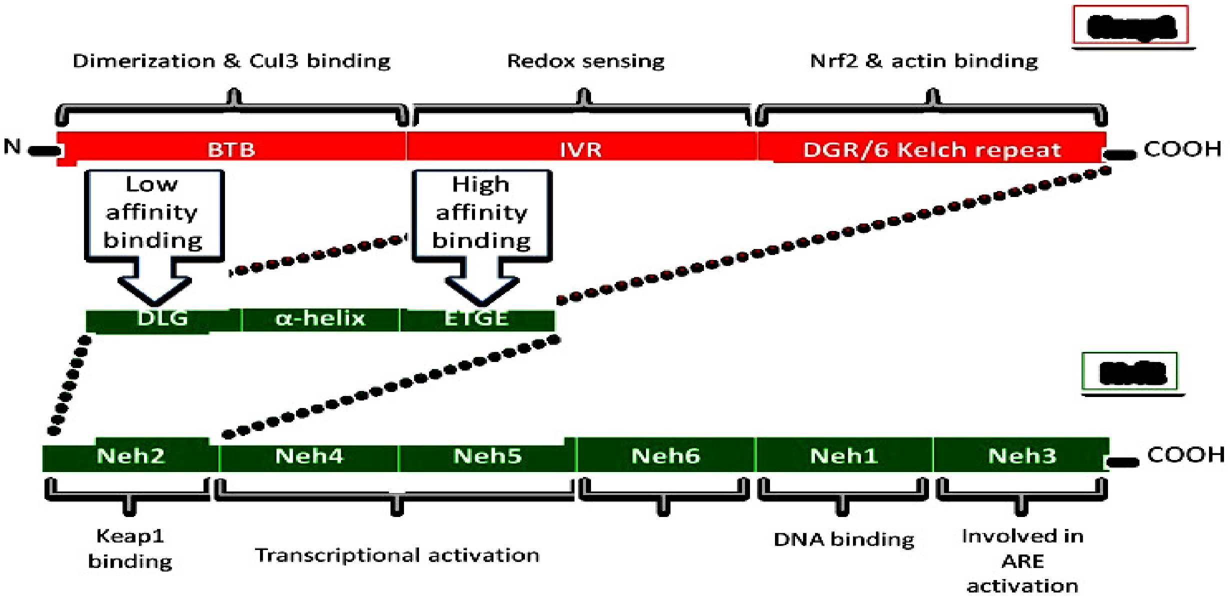

Supplementary Fig. 2 Original blots of hypothyroid and hyperthyroid group of Fig. 1 of the manuscript

<u>**Hypothyroid**</u>

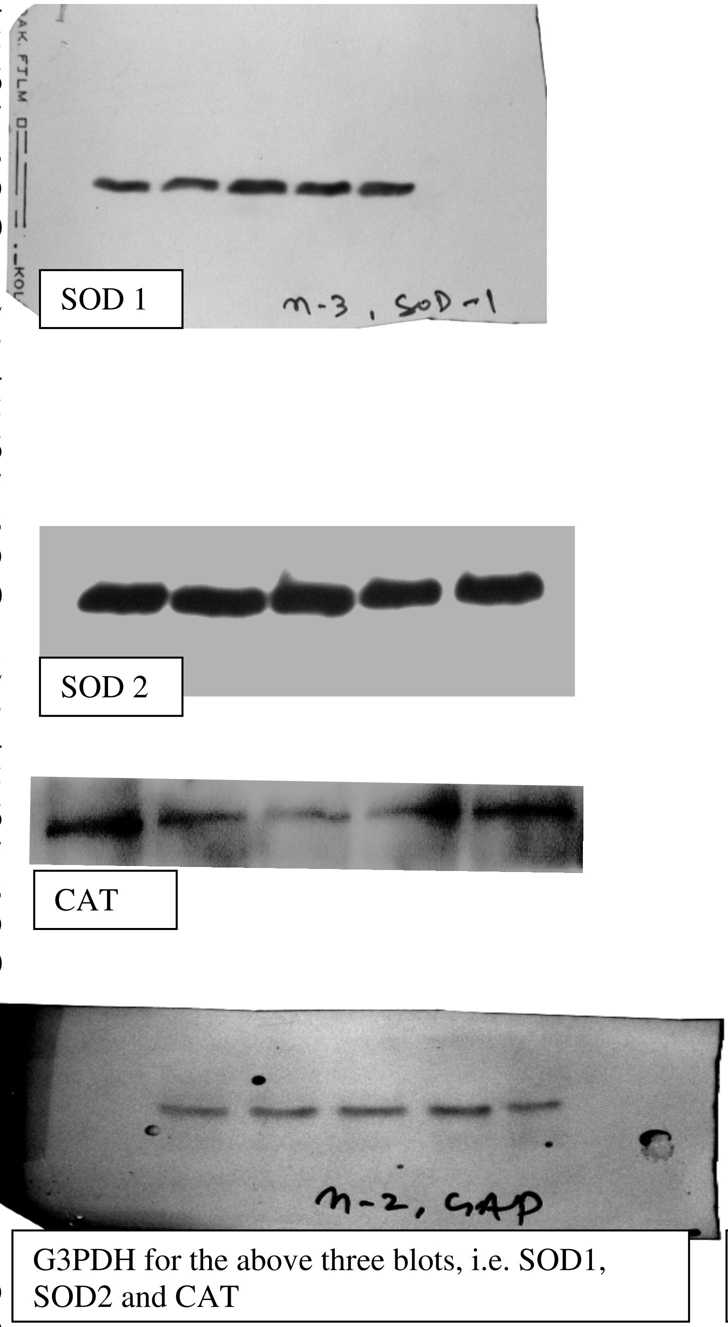

<u>**Hyperthyroid**</u>

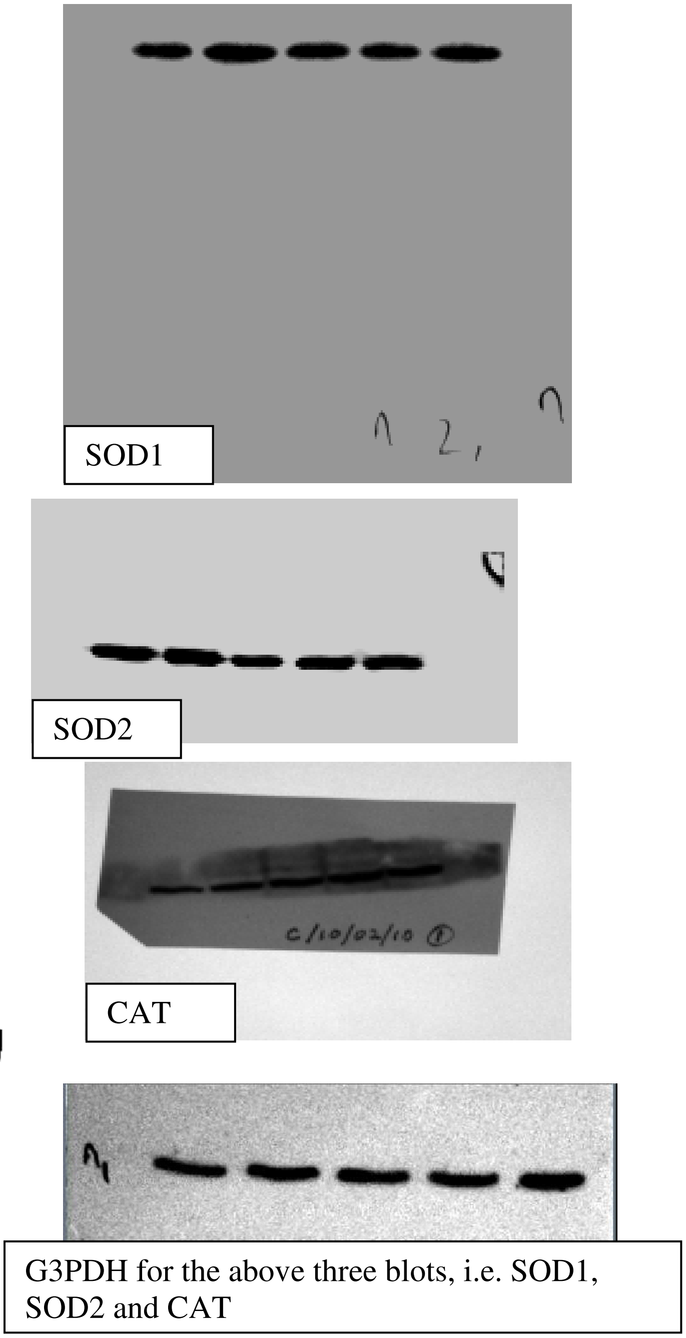

Supplementary Fig. 3 Original blots of hypothyroid and hyperthyroid group of Fig. 2 of the manuscript

<u>Hypothyroid</u> **<u>Hyperthyroid</u>**

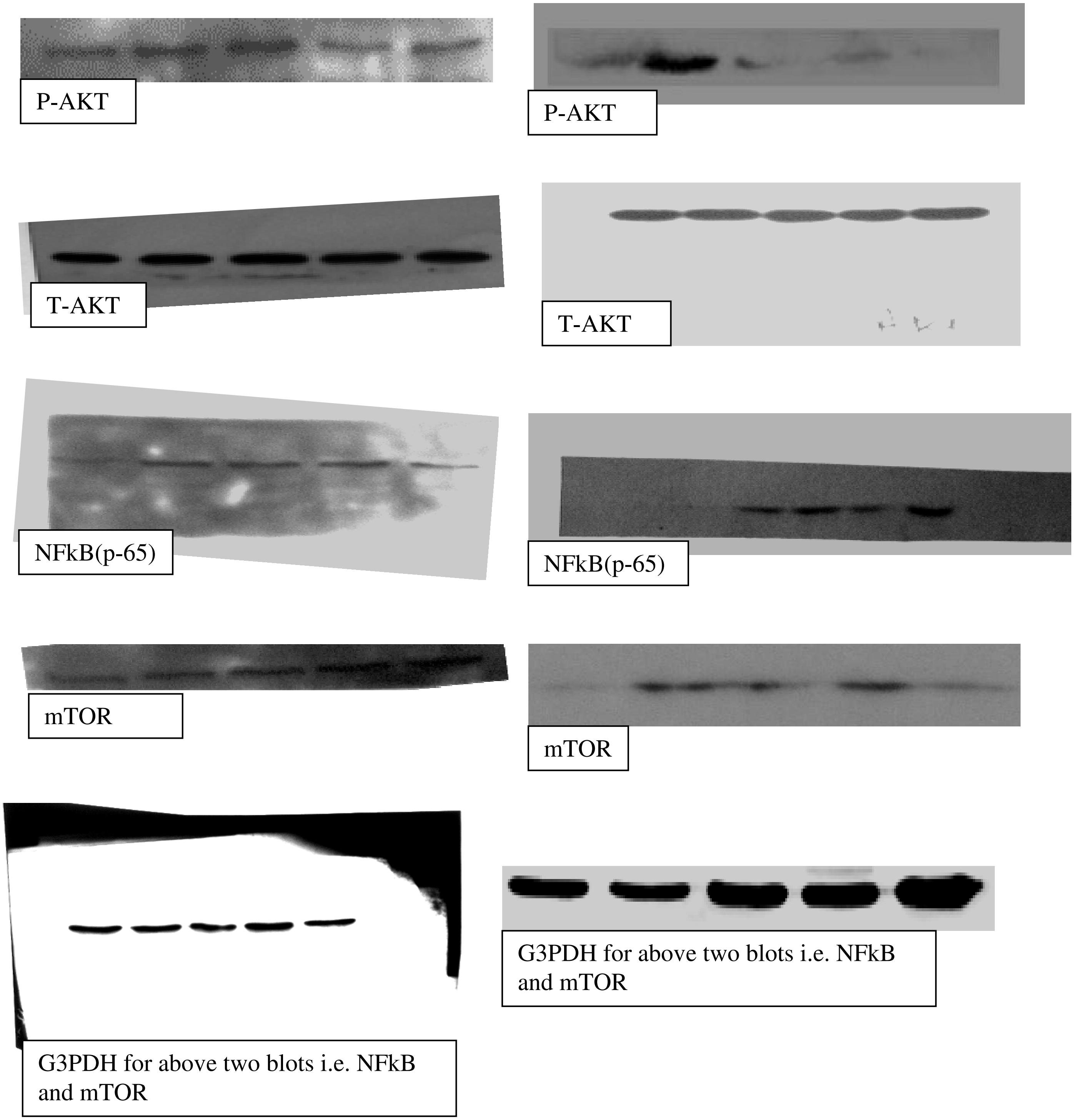

Supplementary Table 1 LSD output for the studies parameters for mean and SD.

Level of trt	N	LPx Mean	LPx SD	SOD Mean	SOD SD	CAT Mean	CAT SD	GPx Mean	GPx SD	GR Mean	GR SD	GHS Mean	GHS SD
**1**	5	3.574	0.576	12.66	1.146	0.204	0.018	0.691	0.056	342.9	19.92	43.14	3.937
**2**	5	3.080	0.239	13.56	0.811	0.218	0.008	0.739	0.066	347.5	13.94	40.38	3.028
**3**	5	5.122	0.637	16.88	1.138	0.162	0.011	0.532	0.076	275.5	35.19	54.38	3.028
**4**	5	4.118	0.396	11.62	0.581	0.188	0.015	0.635	0.046	296.5	20.87	29.14	3.937
**5**	5	3.370	0.450	13.96	1.424	0.236	0.034	0.708	0.105	302.3	10.41	44.74	2.223
**6**	5	2.660	0.305	11.92	0.909	0.192	0.013	0.723	0.037	295.5	21.97	30.74	2.223
**7**	5	3.252	0.459	14.06	1.412	0.208	0.018	0.726	0.092	326.9	20.78	49.56	1.512
**8**	5	2.680	0.349	14.66	1.248	0.210	0.016	0.754	0.063	291.7	14.62	30.75	5.130
**9**	5	3.778	0.266	12.24	1.009	0.250	0.012	0.790	0.108	350.4	15.51	53.82	2.186
**10**	5	2.640	0.404	11.46	0.546	0.208	0.018	0.717	0.029	320.6	12.58	34.50	5.499
